# How cognitively demanding is the urban niche? Reconsidering exaptation and habituation

**DOI:** 10.1007/s10071-025-01965-y

**Published:** 2025-06-17

**Authors:** Lily Johnson-Ulrich, Sofia Forss

**Affiliations:** Department of Evolutionary Biology and Environmental Studies, University of Zurich, Zurich, Switzerland

**Keywords:** Animal urbanization, Animal cognition, Behavioural flexibility, Animal innovation, Problem-solving abilities, Human habituation, Exaptation

## Abstract

Urbanization is hypothesized to create a myriad of cognitive challenges for animals because it creates novel environmental conditions in evolutionary terms. The consensus is that these novel urban challenges act as drivers for increased cognitive abilities. However, scant empirical data validates the idea that urban environments are cognitively demanding relative to native ones. In this short communication we draw the attention to the fact that for some large-brained urban inhabitants the urban environment may instead provide “easy” exploitable niches, where these species can thrive because they already have the necessary cognitive tools in place. As such, evolutionary seen, such species are “exapted” to occupy a less challenging urban niche. As follows, while a species’ cognition may facilitate its persistence under urbanization, it does not necessarily mean that urban populations face selective or developmental drivers for improved cognition in urban living. We further point out the potential bias anthropogenic habituation can bring about when intraspecific comparisons are made between urban and nonurban populations and suggest that researchers must focus on precisely which species-specific aspects of the environment are novel when making predictions about the consequences of urbanization on cognitive traits.

As wildlife globally adjusts to the increasing changes of the Anthropocene, some species not only adapt, but thrive in urban environments. Research into the traits that correlate with persistence under increasing urbanization suggests that the cognitive abilities underlying behavioural flexibility play a key role (Lee and Thornton 2021; Ducatez et al. 2022; Leimar et al. 2024). Here, we refer to behavioural flexibility in the context of problem-solving as *the ability to adaptively adjust behaviour in response to environmental variation* (Lea et al. 2020). Overall, behaviourally flexible species appear more likely to invade novel environments, including urban ones (Sol et al. 2005; Møller 2010; Candolin and Wong 2012; Griffin et al. 2015, 2017b). These results support the Cognitive Buffer Hypothesis (CBH), which suggests that large brains ‘buffer’ the negative consequences of environmental change via increased behavioural flexibility (Sol 2009). As such, behavioural flexibility can result in a more efficient response to urbanization than slower genetic modifications via evolutionary processes.

Following between species comparative results, intraspecific research has begun to compare the cognitive performance of urban versus non-urban populations to identify if urbanization reflects within species cognitive adaptation. The intraspecific approach (Thornton and Lukas 2012; Ashton et al. 2018) is a powerful tool because cognitive abilities tend to develop in response to cognitive challenges and experience (Diamond 2001; Buchanan et al. 2013; Boesch 2020), which allows researchers to more precisely pinpoint what aspects of the environment are cognitively demanding and might ultimately select for cognition. Intraspecific studies almost universally predict that urban populations should have greater behavioural flexibility and score higher on cognitive tests than non-urban populations, as they “learn to adapt” to myriad novel problems in anthropogenic landscapes (Papp et al. 2014; Preiszner et al. 2017; Federspiel et al. 2017; Kang et al. 2018; Batabyal and Thaker 2019; Arnold et al. 2021; Johnson-Ulrich et al. 2021; Vardi and Berger-Tal 2022; Vincze and Kovács 2022; Morton et al. 2023; Biondi et al. 2024; Damas-Moreira et al. 2024). What these novel problems may be has been summarised in other reviews (Lee and Thornton 2021; Sarkar and Bhadra 2022; Mazza and Šlipogor 2024) and includes challenges related to finding food, navigating human grids, avoiding anthropogenic threats, and identifying safe resting sites. Overall, the implicit consensus is that urban habitats pose evolutionarily new cognitive challenges for animals compared to native environments, and the novelty of urban landscapes is seen as the driver of enhanced cognitive capacities, such as behavioural flexibility, in urban relative to native habitats. While this hypothesis is appealing and quite plausible on the surface, intraspecific results regarding the effects of urbanization on cognition have been highly mixed, particularly outside of avian taxa (e.g., Vincze and Kovács 2022).

Before this field of research ‘jumps ahead’ of itself and replicates within species cognitive comparisons across taxa, we believe that greater complexity regarding the interlink between cognition and urbanization must be taken into consideration. We suggest that so far contradictive findings partly derive from a logical non-sequitur; just because behavioural flexibility facilitates adaptation to urban environments in many species, it does not automatically mean that the urban environment must present greater cognitive demands for behavioural flexibility than native or nonurban environments. For most species, there either *is* or *is not* an urban niche available to them based on the *match* or *mismatch* between the urban and native habitat. For species with high behavioural flexibility, afforded by larger brains and cognitive abilities such as innovativeness or inhibitory control, it may be much more feasible to occupy an urban niche. But *how similar* or *different* this niche is from the native niche is likely to be species specific. For example, a dietary generalist may be more likely to find a food source in an urban environment than a specialist species, but rather than the urban niche requiring greater dietary generalism relative to the native habitat, the urban niche may only have one (or reduced diversity of) food source such that a generalist species creates a more specialized population in the urban environment. Likewise, demands for behavioural flexibility may increase or decrease in the urban environment based on a species’ urban niche relative to its native one, irrespective of what traits allowed the species to initially persist in urban environments (see Box 1).

**Box 1:**
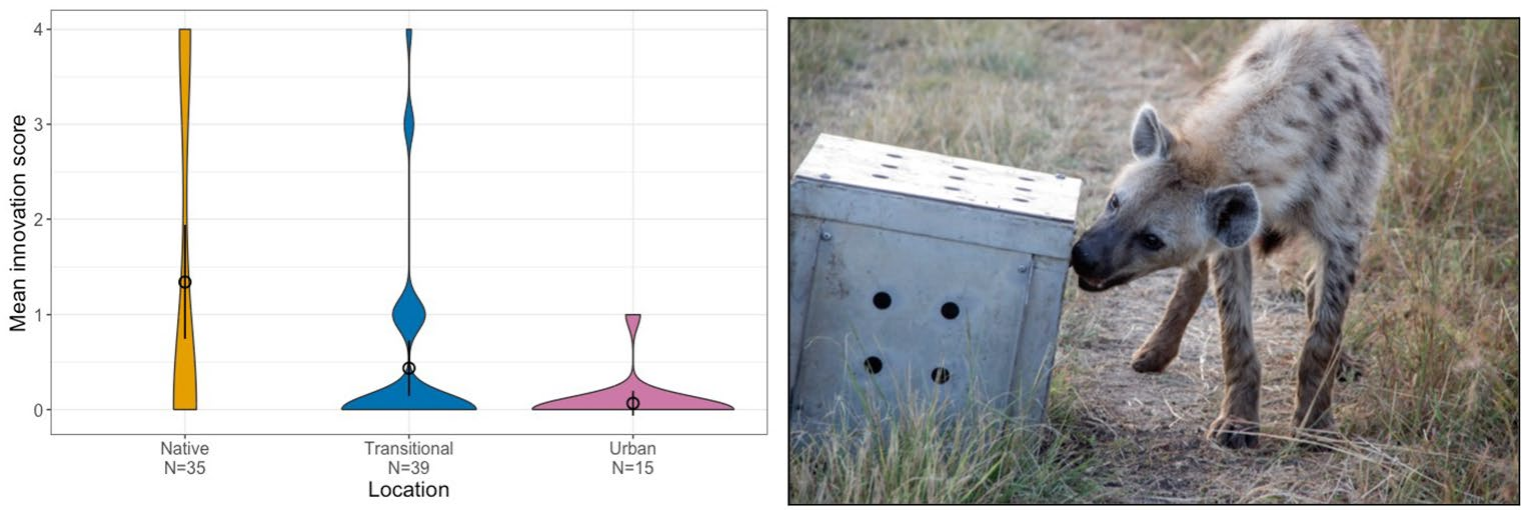
**Reduced innovative problem-solving in urban spotted hyenas, a result of reduced cognitive demands?** It has been suggested that it may not be novel environments themselves that are cognitively challenging, but the transition to novel environments because this is when animals experience the highest level of environmental change for themselves (Wright et al. 2010). Spotted hyenas (*Crocuta crocuta*) are dietary and habitat generalists that live in almost every habitat across sub-Saharan Africa and eat everything from termites to elephants (Holekamp and Dloniak 2010). A study on hyenas compared populations across three different habitats: native, transitional, and long-term, stable urban on an innovative problem-solving test with four different doors by which hyenas could innovate up to four different ways to retrieve the meat from inside (Johnson-Ulrich et al. 2021). In contrast to the predictions that transitional hyenas would be the most innovative, the data showed that the native hyenas scored highest on innovativeness (below: violin plots with means and 95% CI shown by points and bars), while the transitional hyenas did not differ from stable urban hyenas. This suggests that both the transitional and stable urban population may experience reduced demands on innovative abilities possibly because foraging on trash is easier and causes reduced demands for cooperative hunting while also reducing social complexity, or reduced habituation towards tourist vehicles. Figure (left) and image (right) provided by Lily Johnson-Ulrich

## What actually changes under urbanization?

Rather than focusing on environmental novelty and change as a blanket phenomenon affecting behavioural flexibility and cognitive abilities in urban animals, it would be beneficial for future research to focus on how a specific animal’s niche actually changes. A few cognitive studies have made a good start by qualifying the rate of urban change or length of exposure to urbanization (Vrbanec et al. 2021; Johnson-Ulrich et al. 2021; Vardi and Berger-Tal 2022), but we suggest a more tailored approach that examines the urban niche itself (e.g., diet, activity patterns, predators, shelter, exposure to anthropogenic activity). This way, cognitive predictions can be based on the species-specific urban niche, whilst taking developmental trajectories of cognitive traits into consideration. Indeed, one detailed review on animal urbanization concluded that urban habitats generally appeared to be less harsh, more stable, and more predictable than non-urban ones (Griffin et al. 2017a). Thus, for many species urban environments may be *easier* than native ones (e.g., Box 1). We emphasize that the urban niche for many bird species (currently dominating the literature on how cognition links to urbanization) can be widely different than terrestrial mammal species, in part due to greater potential for human-wildlife conflict (Nyhus 2016; Seoraj-Pillai and Pillay 2017). Birds in cities have co-evolved with urban changes, like feeders, and birds also may escape many threats such as cars and human aggression easier through flight than larger terrestrial mammals. Some larger carnivores thrive in urban habitats due to their behavioural adjustment towards nocturnal activities (Gehrt et al. 2010) while other smaller terrestrial animals in gardens may experience relatively similar niches to their native conspecifics (e.g., Damas-Moreira et al. 2024). As such, the predictions for larger-bodied mammalian species such as those in Carnivora or Primates are likely to be different to those of small vertebrates such as birds or lizards, because risk-benefit trade-offs will vary in their available urban niches. We suggest that researchers should not just attempt to more carefully quantify the level of urbanization (Moll et al. 2019; Szulkin et al. 2020), but should quantify, if possible, *niche-specific* variation across urban and nonurban populations. Doing so will also allow researchers to identify *which* changes may be linked to variation in cognition.

### Development and habituation

Given that cognitive abilities are developmentally constructed, especially in larger brained species, careful consideration of how cognitive skills on the individual level are impacted by urban conditions is crucial. For example, motivational traits, such as neophilia and exploration, (Box 2) which impact problem-solving performance, are influenced by experience with anthropogenic materials, food associations, and previous experiences with humans (Chow et al. 2021b; Inzani et al. 2022; Ellington et al. 2024; Lazzaroni et al. 2024; Birchmeier et al. 2023). Therefore, it is highly likely that habituation impacts outcomes in cognitive test situations (Lazure and Weladji 2024). Many urban animals persist on anthropogenic food subsidies and the way researchers commonly test cognition is highly parallel to the way urban animals forage within this niche. As such, cognitive tests may not always represent an unbiased comparison between urban and non-urban animals. For example, foraging from rubbish bins or bird feeders represent quite similar experiences to animals as exposures to cognitive test materials, such as puzzle boxes. Such prior experience, gathered by an urban animal throughout its entire life, must be carefully quantified and considered.

**Box 2:**
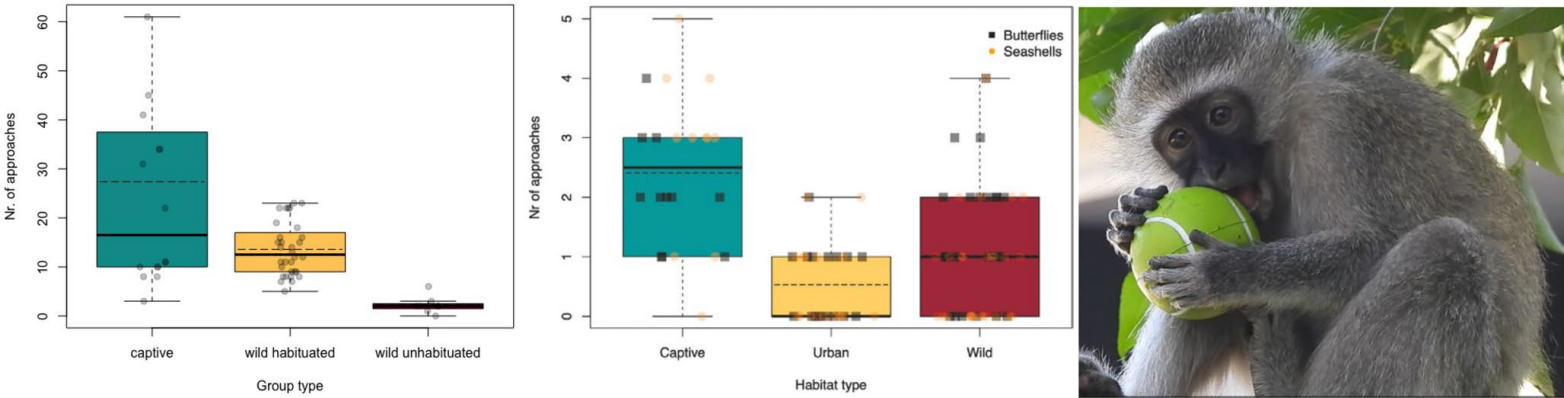
**Human habituation impacts motivation to engage with anthropogenic materials in vervet monkeys.** In vervet monkeys (*Chlorocebus pygerythrus*), habituation to humans raised their expectation of potential to retrieve food rewards and thereby influence their interest in approaching novel artefacts (multiple stimuli including both human-madeand natural items) (Forss et al. 2022). In a sub-sample of the same dataset, urban conspecifics did however not show higher interest in novel artefacts such as rubber butterflies or novel natural items like seashells, likely due to an “over-habituation” towards the diversity of artefacts and human-made objects that are a standard part of their environment (Ellington et al. 2024). Instead, urban monkeys showed selectivity and increased exploration of food associated anthropogenic items, thus urbanization has made them “anthrophilic” (interested in items associated with human food sources) rather than generally neophilic (interested in novel items) (Ellington et al. 2024). Figure (left) reproduced under CC BY 4.0 from (Forss et al. 2022) and figure (right) under OUP license 5955320465856 from (Ellington et al. 2024). Photograph by Urban Vervet Project

### Expated for urban environments?

Ultimately, cognitive capacities underlying behavioural flexibility are just a few among many traits that may ‘exapt’ species to urbanization. Here, we use the word ‘exaptation’ to draw attention to the possibility that pre-existing species’ level traits such as behavioural flexibility and learning facilitate the invasion of (or persistence in) urban environments, especially in large-brained animals. Indeed, behavioural modifications (such as integrating a new food source) that occurs when a species adjusts to the anthropogenic changes are linked to the species’ general learning abilities. As such, the mechanism of adaptation (here learning capacity) may be at the individual level the very same as in the native habitat. In other words, large brained species that use their learning capacities to master extractive foraging challenges in their natural environment, may be well prepared to learn extractive foraging challenges in the urban habitat. Thus, environmental inputs during development will utilize those cognitive capacities regardless of the individual living in the native or an urban niche. I.e., while many cognitive challenges are evolutionary novel at the species-level, to the developing individual the challenges are within the species repertoire and may even be less cognitively demanding. Thus, the “exaptation” view considers that a species’ may already be cognitively equipped for the urban environment (Kark et al. 2007; Sol 2008; Polley and Lill 2021; Chow et al. 2021a), and we must therefore not necessarily expect the urban population to show superior abilities (Box 3).

**Box 3:**
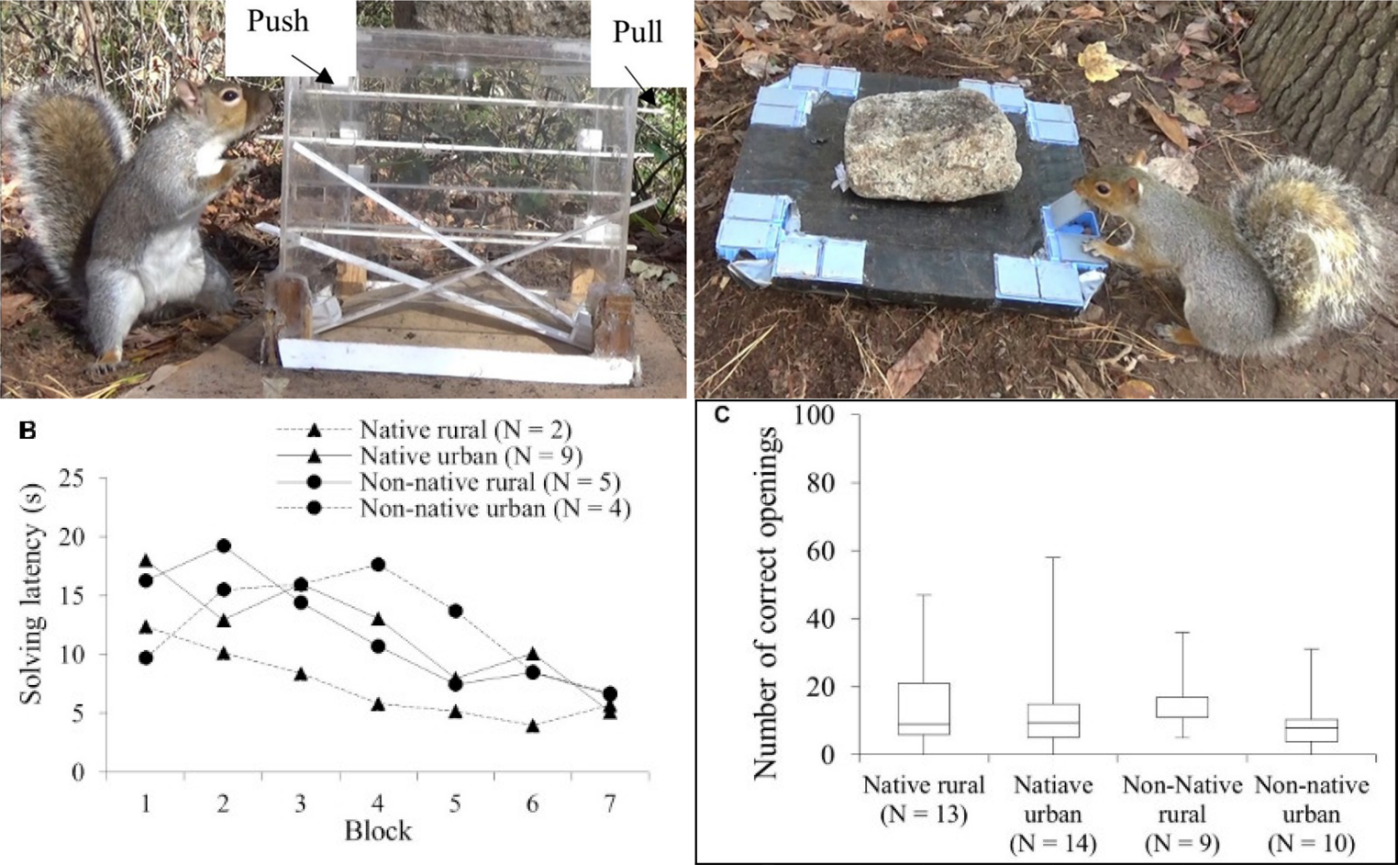
**Pre-adaptative cognitive ability in squirrels facilitates invasion of urban and non-native habitats.** Eastern gray squirrels (*Sciurus carolinensis*) are both invasive outside their range and successful urban adapters while showing superior problem-solving abilities compared to other squirrel species (Chow et al. 2018). To investigate whether novel environments select for enhanced problem-solving, researchers compared the cognition of gray squirrels in both native and non-native, urban and nonurban populations but found that problem-solving performance across a range of cognitive tasks was largely similar across all four populations (Chow et al. 2021a). They concluded that gray squirrels’ superior problem-solving abilities likely pre-adapt (i.e., exapt) them for living in novel environments and that these novel environments subsequently exert only minimal effects on cognitive abilities. Below: No significant differences were found across populations for solving latency across time in an extractive foraging task where squirrels had to push or pull a lever to retrieve containers with nuts (left) and number of correct openings in a spatial-learning task where squirrels had to remember the location of containers with accessible nuts in a small array (right). ‘Blocks” represent groups of 5 successful trials over time. N indicates the number of squirrels. Figures reproduced from (Chow et al. 2021a) under a CC BY license

### Concluding remarks

In sum, we hope this short communication will benefit researchers to finetune cognitive predictions based on well-validated socio-ecological hypotheses that reflect the differences between evolutionary patterns (between species variation) and developmental trajectories (within species variation). We reiterate that we are not suggesting that animals do not use their cognitive capacities in urban environments in adaptive or even novel ways, only that when comparing across urban versus nonurban populations one should identify to what extent the urban environment is more or less cognitively demanding than native environments. In short, to understand the role cognition plays in animal urbanization, we need individualized approaches with nuanced predictions at the species’ level based on quantifiable differences between urban and nonurban niches.

## Data Availability

No datasets were generated or analysed during the current study.

## References

[CR1] Arnold S, Weaver MJ, McGraw KJ (2021) Ornamental plumage coloration interacts with habitat urbanization to predict problem-solving in the house finch hemorhous Mexicanus. Acta Ornithol 56. 10.3161/00016454ao2021.56.1.003

[CR2] Ashton BJ, Thornton A, Ridley AR (2018) An intraspecific appraisal of the social intelligence hypothesis. Philosophical Trans Royal Soc B: Biol Sci 373:20170288. 10.1098/rstb.2017.0288 10.1098/rstb.2017.0288 PMC6107571 30104433

[CR3] Batabyal A, Thaker M (2019) Lizards from suburban areas learn faster to stay safe. Biol Lett 15:20190009. 10.1098/rsbl.2019.0009 30958137 10.1098/rsbl.2019.0009 PMC6405467

[CR4] Biondi LM, Medina A, Bonetti EA et al (2024) Cognitive flexibility in a generalist raptor: a comparative analysis along an urbanization gradient. Behav Ecol 35. 10.1093/beheco/arae025

[CR400] Birchmeier K, Johnson-Ulrich L, Stein J et al (2023) The Role of Umwelt in Animal Curiosity: A Within and Between Species Comparison of Novelty Exploration in Mongooses. Anim Behav Cognit 10(4):329ȃ354. 10.26451/abc.10.04.03.2023

[CR5] Boesch C (2020) Mothers, environment, and ontogeny affect cognition. Anim Behav Cogn 7:1–16. 10.26451/abc.07.03.01.202032626823

[CR6] Buchanan KL, Grindstaff JL, Pravosudov VV (2013) Condition dependence, developmental plasticity, and cognition: implications for ecology and evolution. Trends Ecol Evol 28:290–296. 10.1016/j.tree.2013.02.004 23518414 10.1016/j.tree.2013.02.004 PMC3640828

[CR7] Candolin U, Wong BBM (2012) Behavioural responses to a changing world: mechanisms and consequences. Oxford University Press

[CR9] Chow PKY, Lurz PWW, Lea SEG (2018) A battle of wits? Problem-solving abilities in invasive Eastern grey squirrels and native Eurasian red squirrels. Anim Behav 137:11–20. 10.1016/j.anbehav.2017.12.022

[CR8] Chow PKY, Clayton NS, Steele MA (2021a) Cognitive performance of wild Eastern Gray squirrels (Sciurus carolinensis) in rural and urban, native, and Non-native environments. Front Ecol Evol 9:1–15. 10.3389/fevo.2021.615899

[CR10] Chow PKY, Uchida K, von Bayern AMP, Koizumi I (2021b) Characteristics of urban environments and novel problem-solving performance in Eurasian red squirrels. Proceedings of the Royal Society B: Biological Sciences 288:rspb.2020:2832. 10.1098/rspb.2020.2832 10.1098/rspb.2020.2832 PMC8059950 33784870

[CR11] Damas-Moreira I, Szabo B, Drosopoulos G et al (2024) Smarter in the city? Lizards from urban and semi-natural habitats do not differ in a cognitive task in two syntopic species. Curr Zool 70:361–370. 10.1093/cz/zoae010 39035752 10.1093/cz/zoae010 PMC11255991

[CR12] Diamond MC (2001) Response of the brain to enrichment. Acad Bras Cienc 73:210–220. 10.1590/s0001-37652001000200006 10.1590/s0001-37652001000200006 11404783

[CR13] Ducatez S, DeVore JL, Whiting MJ, Audet J-N (2022) Editorial: cognition and adaptation to urban environments. Front Ecol Evol 10:721–729. 10.3389/fevo.2022.953494

[CR14] Ellington L, Mercier S, Motes-Rodrigo A et al (2024) Urbanization does not increase ‘object curiosity’ in Vervet monkeys, but semi-urban individuals selectively explore food related anthropogenic items. 10.1093/cz/zoae022. Curr Zool zoae022 10.1093/cz/zoae022 PMC11255996 39035753

[CR15] Federspiel IG, Garland A, Guez D et al (2017) Adjusting foraging strategies: a comparison of rural and urban common Mynas (Acridotheres tristis). Anim Cogn 20:65–74. 10.1007/s10071-016-1045-7 27778195 10.1007/s10071-016-1045-7

[CR16] Forss SIF, Motes-Rodrigo A, Dongre P et al (2022) Captivity and habituation to humans Raise curiosity in Vervet monkeys. Anim Cogn 25:671–682. 10.1007/s10071-021-01589-y 34855018 10.1007/s10071-021-01589-y PMC9107434

[CR17] Gehrt S, Riley SPD, Cypher BL (eds) (2010) Urban carnivores. Johns Hopkins University

[CR18] Griffin AS, Guez D, Federspiel IG et al (2015) In: Sol D (ed) Invading new environments: a mechanistic framework linking motor diversity and cognitive processes to invasion success. Biological invasions and behavior. JS Weis, Cambridge, UK: Cambridge University Press

[CR19] Griffin AS, Netto K, Peneaux C (2017a) Neophilia, innovation and learning in an urbanized world: a critical evaluation of mixed findings. Curr Opin Behav Sci 16:15–22. 10.1016/j.cobeha.2017.01.004

[CR20] Griffin AS, Tebbich S, Bugnyar T (2017b) Animal cognition in a human-dominated world. Anim Cogn 20:1–6. 10.1007/s10071-016-1051-9 27848045 10.1007/s10071-016-1051-9

[CR21] Holekamp KE, Dloniak SM (2010) Intraspecific variation in the behavioral ecology of a tropical carnivore, the spotted hyena. In R. Macedo (Ed.), Advances in the Study of Behavior (Vol. 42, pp. 189–229). Academic Press. 10.1016/S0065-3454(10)42006-9

[CR22] Inzani EL, Kelley LA, Boogert NJ (2022) Object neophilia in wild herring gulls in urban and rural locations. J Avian Biol 1–11. 10.1111/jav.03028

[CR23] Johnson-Ulrich L, Yirga G, Strong RL, Holekamp KE (2021) The effect of urbanization on innovation in spotted hyenas. Anim Cogn 24:1027–1038. 10.1007/s10071-021-01494-4 33687598 10.1007/s10071-021-01494-4

[CR24] Kang F, Goulet CT, Chapple DG (2018) The impact of urbanization on learning ability in an invasive Lizard. Biol J Linn Soc 123:55–62

[CR25] Kark S, Iwaniuk A, Schalimtzek A, Banker E (2007) Living in the city: can anyone become an urban exploiter? J Biogeogr 34:638–651. 10.1111/j.1365-2699.2006.01638.x

[CR26] Lazure L, Weladji RB (2024) Exposure to humans and task difficulty levels affect wild raccoons (Procyon lotor) learning. Behav Ecol 35:arae046. 10.1093/beheco/arae046 38912327 10.1093/beheco/arae046 PMC11190377

[CR27] Lazzaroni M, Brogi R, Napolitano V et al (2024) Urbanization does not affect red foxes’ interest in anthropogenic food, but increases their initial cautiousness. 10.1093/cz/zoae023. Curr Zool zoae023 10.1093/cz/zoae023 PMC11255992 39035755

[CR28] Lea SEG, Chow PKY, Leaver LA, McLaren IPL (2020) Behavioral flexibility: A review, a model, and some exploratory tests. Learn Behav. 10.3758/s13420-020-00421-w 32043268 10.3758/s13420-020-00421-w PMC7082303

[CR29] Lee VE, Thornton A (2021) Animal cognition in an urbanised world. Front Ecol Evol 9:1–20. 10.3389/fevo.2021.633947 10.3389/fevo.2021.633947 PMC7611524 34409044

[CR30] Leimar O, Quiñones AE, Bshary R (2024) Flexible learning in complex worlds. Behav Ecol 35:1–12. 10.1093/beheco/arad109 10.1093/beheco/arad109 PMC10756056 38162692

[CR31] Mazza V, Šlipogor V (2024) Behavioral flexibility and novel environments: integrating current perspectives for future directions. Curr Zool 70:304–309. 10.1093/cz/zoae029 39035762 10.1093/cz/zoae029 PMC11255986

[CR32] Moll RJ, Cepek JD, Lorch PD et al (2019) What does urbanization actually mean? A framework for urban metrics in wildlife research. J Appl Ecol 56:1289–1300. 10.1111/1365-2664.13358

[CR33] Møller AP (2010) Interspecific variation in fear responses predicts urbanization in birds. Behav Ecol 21:365–371. 10.1093/beheco/arp199

[CR34] Morton FB, Gartner M, Norrie E et al (2023) Urban foxes are bolder but not more innovative than their rural conspecifics. Anim Behav 203:101–113. 10.1016/j.anbehav.2023.07.003

[CR35] Nyhus PJ (2016) Human-Wildlife conflict and coexistence. Annu Rev Environ Resour 41:143–171. 10.1146/annurev-environ-110615-085634

[CR36] Papp S, Vincze E, Preiszner B et al (2014) A comparison of problem-solving success between urban and rural house sparrows. Behav Ecol Sociobiol 69:471–480. 10.1007/s00265-014-1859-8

[CR37] Polley E, Lill A (2021) Foraging of Sulphur-crested cockatoos: examining the roles of preadaptation, behavioural flexibility and interspecific competition in urban dwelling. Corella 45:7–16

[CR38] Preiszner B, Papp S, Pipoly I et al (2017) Problem-solving performance and reproductive success of great Tits in urban and forest habitats. Anim Cogn 20:53–63. 10.1007/s10071-016-1008-z 27294267 10.1007/s10071-016-1008-z

[CR39] Sarkar R, Bhadra A (2022) How do animals navigate the urban jungle? A review of cognition in urban-adapted animals. Curr Opin Behav Sci 46:101177. 10.1016/j.cobeha.2022.101177

[CR40] Seoraj-Pillai N, Pillay N (2017) A meta-analysis of human-wildlife conflict: South African and global perspectives. Sustain (Switzerland) 9:1–21. 10.3390/su9010034

[CR42] Sol D (2008) Do successful invaders exist?? Pre-Adaptations to novel environments in terrestrial vertebrates. In: Nentwig W (ed) Biological invasions. Ecological studies (Analysis and Synthesis), vol 193. Springer, Berlin, Heidelberg, pp 127–141

[CR41] Sol D (2009) The cognitive-buffer hypothesis for the evolution of large brains. In: Dukas R, Ratcliffe JM (eds) Cognitive ecology II. The University of Chicago Press, Chicago, IL, pp 111–134

[CR43] Sol D, Duncan RP, Blackburn TM et al (2005) Big brains, enhanced cognition, and response of birds to novel environments. Proc Natl Acad Sci U S A 102:5460–5465. 10.1073/pnas.0408145102 15784743 10.1073/pnas.0408145102 PMC556234

[CR44] Szulkin M, Garroway CJ, Corsini M et al (2020) How to quantify urbanization when testing for urban evolution. Urban Evolutionary Biology 13:1861–1876

[CR45] Thornton A, Lukas D (2012) Individual variation in cognitive performance: developmental and evolutionary perspectives. Philos Trans R Soc Lond B Biol Sci 367:2773–2783. 10.1098/rstb.2012.0214 22927576 10.1098/rstb.2012.0214 PMC3427550

[CR46] Vardi R, Berger-Tal O (2022) Environmental variability as a predictor of behavioral flexibility in urban environments. Behav Ecol 33:573–581. 10.1093/beheco/arac002

[CR47] Vincze E, Kovács B (2022) Urbanization’s effects on problem solving abilities: A Meta-Analysis. Front Ecol Evol 10:1–8. 10.3389/fevo.2022.834436

[CR48] Vrbanec L, Matijević V, Guenther A (2021) Enhanced problem-solving ability as an adaptation to urban environments in house mice. Proc Royal Soc B: Biol Sci 288:20202504. 10.1098/rspb.2020.2504 10.1098/rspb.2020.2504 PMC7934975 33593181

[CR49] Wright TF, Eberhard JR, Hobson EA et al (2010) Behavioral flexibility and species invasions: the adaptive flexibility hypothesis. Ethol Ecol Evol 22:393–404. 10.1080/03949370.2010.505580

